# Initial application of transanal endoscopic microsurgery for high-risk lower rectal gastrointestinal stromal tumor after imatinib mesylate neoadjuvant chemotherapy: A case report

**DOI:** 10.1097/MD.0000000000007538

**Published:** 2017-07-21

**Authors:** Qiaofei Liu, Guangxi Zhong, Weixun Zhou, Guole Lin

**Affiliations:** a Department of General Surgery; b Department of Ultrasound; c Department of Pathology, Peking Union Medical College Hospital, Peking Union Medical College and Chinese Academy of Medical Sciences, Beijing, China.

**Keywords:** gastrointestinal stromal tumor, imatinib mesylate, lower rectal, neoadjuvant therapy, transanal endoscopic microsurgery

## Abstract

**Rationale:**

The lower rectal gastrointestinal stromal tumor (GIST) is a rare entity and warrants special attentions because of the considerations of preserving of anal and urinal functions. Neoadjuvant therapy with imatinib mesylate (IM) has achieved great success in GIST, which potentially extends the applications of function-preserving minimally invasive surgical procedures. Transanal endoscopic microsurgery (TEM) is a well-developed minimally invasive technique for benign tumors in lower rectum. Herein, we reported the initial application of TEM for high risk GIST after IM treatment.

**Patient concerns:**

A 52-year-old woman suffered mild lower abdominal pain and perianal discomfort. Physical examination found a soft mass 4 cm far away from anal verge. Rectal MRI and transrectal ultrasound (TRUS) showed that there was a 1.9 × 1.6 cm submucosal mass in the lower rectum. The incisional biopsy was performed and the pathological result reported it was a high-risk GIST.

**Diagnoses:**

High-risk lower rectal GIST.

**Interventions:**

IM was given for neoadjuvant therapy. Then TEM was adopted to resect the residual tumor. IM was restored 4 weeks after surgery.

**Outcomes:**

The final pathological results reported the margin was clear. After an 18-month follow up, no recurrence and metastasis was found and the patient had a satisfactory anal and urinal functions.

**Lessons:**

TEM in combination with IM could be a practical strategy for the high-risk lower rectal GIST simultaneously to achieve curative resection and to preserve the anal and urinal functions that can significantly improve the life quality of the patients.

## 1 Introduction

Gastrointestinal stromal tumor (GIST) is the largest group of mesenchymal tumors in the gastrointestinal tract, most of them are located in stomach, only less than 5% are located in large bowel, and the tumors in lower rectum are less than 1%.^[1]^ Although lower rectal GIST is a rare entity, it warrants especial considerations of preserving of key structures. During the last decade, imatinib mesylate (IM) has shown dramatically effects on recurrent or metastatic GIST that has changed the perioperative strategies of GIST.^[2,3]^ Different from adenocarcinoma, GIST is less invasive and always shows much better prognosis.^[4]^ Recent years, several cases have reported that IM neoadjuvant therapy significantly decreased the tumor size of GIST in rectum that permits the possibility of function-preserving operations.^[5–7]^ The emerging neoadjuvant therapy of IM may extend the applications of minimally invasive surgical procedure for this kind of tumors. Transanal endoscopic microsurgery (TEM) was developed by Professor Gerhard Buess 30 years ago.^[8]^ It is a safe, well-established minimally invasive technique to remove lesions in the middle-lower rectum via the anus. Compared with the conventional transanal procedure, TEM is more precise with magnified high-resolution surgical field and can reach a upper distance ((8-12 vs 12-15 cm).^[9]^ TEM was initially designed to treat middle-lower rectal small benign lesions. Recent years, it has also been applied to treat early rectal cancers,^[10]^ carcinoid,^[11]^ and even melanoma,^[12]^ and the results seem satisfactory; however, seldom cases of the applications of TEM in GIST have been reported. The potential powerful effects of IM on GIST before surgery to reduce the tumor size may facilitate the application of TEM in GIST. Up to now, a few cases of TEM in combination with IM to treat GIST has been reported.^[13,14]^ In this study, we initially reported a case of high-risk lower rectal GIST treated by TEM in combination with IM. After neoadjuvant therapy with IM, the tumor shrank significantly, and then TEM was adopted to resect the tumor. The perioperative period was uneventful. The IM treatment also significantly reduced the kit-67 index and mitotic activities. After an 18-month follow up, no local recurrence or distant metastasis was found, and the patient had satisfactory anal and urinal function.

## 2 Case presentation

**Figure 1:**
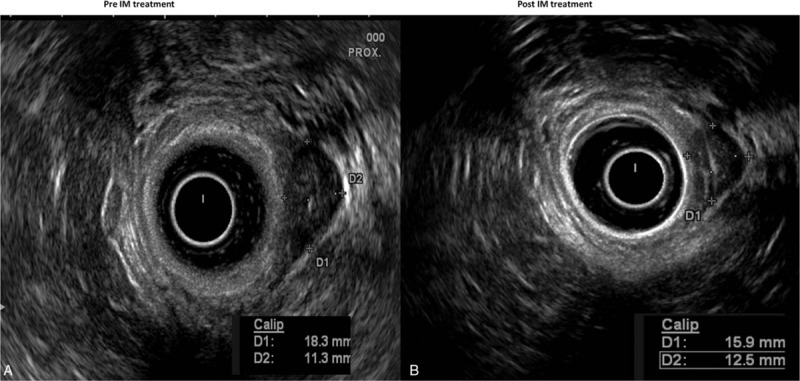
The images of the tumor in TRUS: A, pre-IM treatment; (B) post-IM treatment (9 months later).

**Figure 2:**
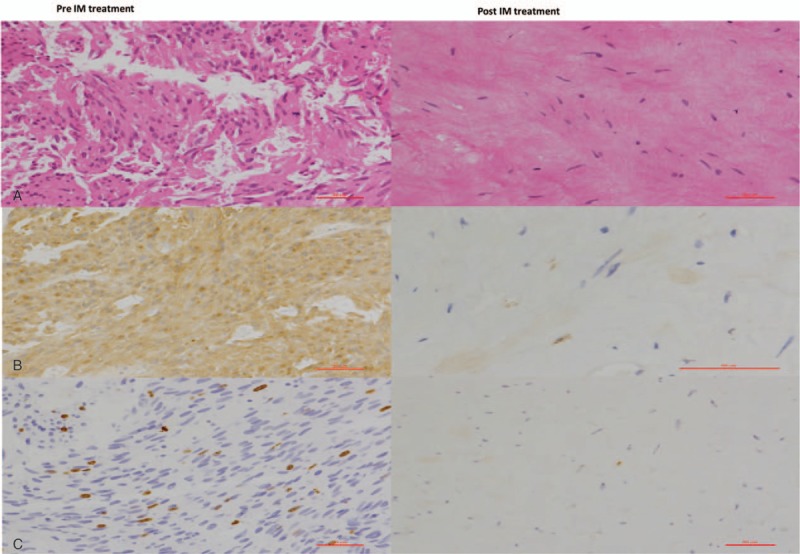
The pathological images of the tumor before and after IM treatment: A, the hematoxylin and eosin staining; B, the IHC staining of CD117; C, the IHC staining of Kit-67.

**Figure 3:**
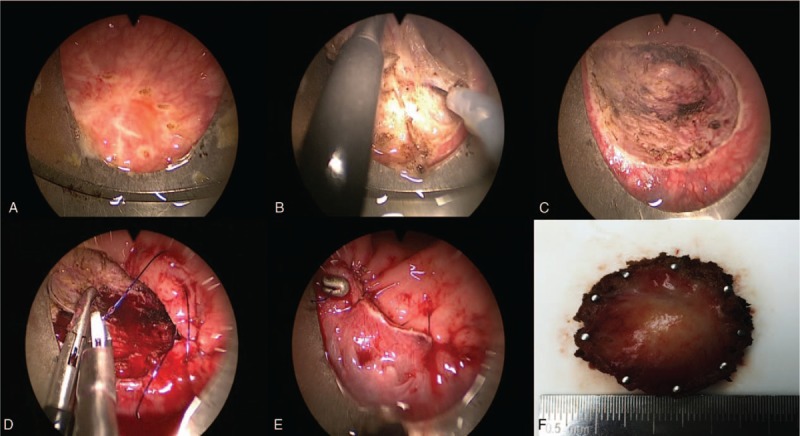
The procedures of TEM: A, mark the tumor margin; B, resect with dot electrical coagulator; C, en bloc full-thickness resection; D, running suture with 4–0 absorbable line; E, the structured incision; F, the en bloc resected tumor.

In April 2014, this 52-year-old female patient began to suffer mild lower abdominal pain and perianal discomfort. And then she visited the outpatient clinic of general surgery. The routine physical examination found a soft mass in the lower rectum 4 cm far away anal verge. The tumor makers, including, CEA, CA199, and CA242 were scanned and all of them were normal. A rectal MRI and transrectal ultrasound (TRUS) were performed. The results showed that there was a 1.9 × 1.6 cm submucosal mass located within the rectal wall 4 to 5Ycm away from anal verge (Fig. 1). The mass had a clear margin that indicated it could be a benign tumor. She underwent total hysterectomy because of hysteromyoma in 2010. She had no history of smoking and abuse of alcohol. She did not have a family history of colorectal cancer or any other hereditary colorectal disease. In May 2014, the incisional biopsy under rectoscopy was performed. The pathological results reported it was a spindle cell tumor. The immunohistochemical (IHC) staining showed the tumor cells were CD117 (+), DOG-1 (−), and the Kit-67 index was 15%. The mitotic count was 5/10 HPF (Fig. 2). The diagnosis of high-risk GIST was verified. Then abdominal and pelvic intravenous enhanced computed tomography (CT) scan was performed and no other lesion was found. Because of the extremely low location from anal verge, the high-risk level of tumor and the consideration of complete preserving of the anal and urinal function, the patient underwent IM neoadjuvant therapy with the aim to downstage of the tumor. Oral 400 mg of IM per day was prescribed to her. Three months later, another TRUS was performed; however, the result showed the tumor did not shrink. Six months later, the TRUS showed the tumor slightly shrank. Nine months later, the TRUS showed the tumor significantly shrank to 1.5 cm × 1.2 cm (Fig. 1). Twelve months later, the TRUS showed the tumor did not change any more, so the surgery was performed. Under general anesthesia, with the patient in supine position, the TEM was adopted to en bloc resect the full thickness of the rectum (Fig. 3). The operational time was 30 minutes and 2 days after operation, the patient was discharged. The postoperative period was uneventful. The final pathological results reported that the tumor was 1.8 cm × 1.2 cm in diameter with extensive hyaline degeneration. The kit-67 index was 1% and the mitotic count was less than 2/50HPF (Fig. 2). IM was restored 4 weeks later. After an 18-month follow up, no local recurrence and distant metastasis was observed and the patient had satisfactory anal and urinal functions. The patient signed the informed consent and the clinical research ethics committee of Peking Union Medical College Hospital approved this study.

## 3 Discussion

The balance of radical resection of the tumor and the preserving of the anal and urinal functions can be the most important consideration to treat lower rectal tumors. Because of the narrow surgical field and close distance to anal verge, it is extremely challenging to achieve radical resect with complete function-preserving and the abdominal peritoneum resection (APR) is the traditional predominant choice. Recent years, for the advanced lower rectal adenocarcinoma, neoadjuvant chemoradiotherapy has been recommended as the standard regimen to downstage of the tumor that can facilitate curative resection with preserving of anal and urinal functions.^[15]^ TEM was first developed by Professor Gerhard Buess almost 30 years ago in an animal model.^[8]^ And then several modifications have been made to improve the visibility by employing superior optics, carbon dioxide-induced rectal distention, and longer instruments. Nowadays, it has been widely applied and has shown promising effects to treat benign middle-lower rectal lesions, early lower rectal cancer, and some other lower malignant tumors. With the aid of advanced instruments, TEM enables full-thickness excision and accurate resection with sufficient margins. Compared with the APR and some other function-preserving procedures, TEM is much more minimally invasive with less morbidity and better life quality for selective patients.^[16]^ Lower rectal GIST is a rare entity consisting of less than 1% of GIST originated from gastrointestinal tract. GIST is a kind of heterogeneous tumor that should be stratified to different risk levels, ranging from very low risk, low risk, intermediate risk to high risk, according to the tumor size, tumor location, and activity of mitosis.^[17]^ IM, a small molecule inhibitor of the oncoprotein Kit and platelet-derived growth factor receptor alpha (PDGFRA), has been recommended to be the standard treatment for recurrent and metastatic GIST. Neoadjuvant therapy with IM for lower rectal GIST has been seldom reported.^[5]^ This tumor was located in lower rectum and the mitotic count was up to 5/10HPF, it was considered to be high-risk level. So we prescribed 400 mg IM per day for the patient before surgery. And then intense follow up by TRUS was performed with an interval of 3 months. Six months after IM treatment, the tumor began to shrink. And 12 months later, the tumor did not change any more, so the operation was performed. Up to now, the duration of IM treatment before surgery is controversial without international consensus.^[18]^ The resistance to imatinib of GIST always occurs 2 years after treatment because of the secondary mutation of PDGFRA. In this case, 6 months after treatment, the tumor began to shrink; 9 months later, the tumor still responded well to the treatment; till 12 months, it did not change any more, so we thought it should be the best chance to perform operation. In the largest series of rectal GIST, most of the tumors were treated by transanal local resection or APR, no one adopted the method of TEM.^[5]^ Compared the traditional transanal local excision, TEM has many advantages to achieve radical resection, in aspects of magnified surgical filed, high resolution view, precise coagulation, and sucking instrument.^[19]^ Although a few cases of TEM to treat GIST were reported, considering of the small tumor size, high mitotic activity and close distance to anal verge, we performed TEM for her. The operation was smooth, and the postoperative period was uneventful. The final pathological result reported the margin was clear and the Kit-67 index was only 1% and the mitotic count was less than 2/50HPF. Some other cases also reported that imatinib treatment could reduce the Kit-67 index and the activity of mitosis of GIST.^[5]^ One month after operation, IM was restored. After an 18-month follow up, no recurrence or metastasis was found and the patient has satisfactory anal and urinal functions. This case provided a new strategy consisting of TEM in combination with IM to treat high-risk lower rectal GIST, especially when the tumor is small and response well to IM. However, for further validation of the effects of this new strategy, prospective studies are needed in the future.

## 4 Conclusion

In summary, lower rectal GIST is a rare entity that warrants special attentions of preserving anal and urinal functions. In this case, TEM in combinations with IM neoadjuvant therapy achieved curative resection of the tumor, satisfactory anal and urinal function and good long-term prognosis. For the lower rectal GIST, especially when the tumor is small and response well to IM, TEM in combination with IM neoadjuvant therapy could be the initial attempt to achieve simultaneous radical resection of the tumor and satisfactory anal and urinal function-preserving; moreover, prospective studies with a good number of such patients from multicenters are needed to further validate its effects.
